# Uterine Inflammatory Myofibroblastic Tumor: A Case Report

**DOI:** 10.7759/cureus.106220

**Published:** 2026-03-31

**Authors:** Maria Inês Ribeiro, Joana Ferreira, Teresa Margarida Cunha

**Affiliations:** 1 Radiology, Instituto Português de Oncologia de Lisboa Francisco Gentil, Lisbon, PRT; 2 Pathology, Instituto Português de Oncologia de Lisboa Francisco Gentil, Lisbon, PRT

**Keywords:** abnormal uterine bleeding, anaplastic lymphoma kinase, inflammatory myofibroblastic tumor, uterine sarcoma, uterus

## Abstract

Uterine inflammatory myofibroblastic tumor (IMT) is a rare type of mesenchymal tumor that usually follows an indolent course, although it can behave aggressively in a minority of cases. The most common clinical presentations are abnormal uterine bleeding and pelvic discomfort. Radiographic features are non-specific, typically showing a myometrial mass with heterogeneous enhancement and, occasionally, infiltrative margins. Histologically, IMTs are composed of spindle myofibroblasts in a myxoid to compact stroma with an inflammatory infiltrate, and they often exhibit anaplastic lymphoma kinase (ALK) gene fusions and ALK positivity. The main treatment approach is surgical excision, with ALK inhibitors as a therapeutic option in selected cases.

## Introduction

Uterine inflammatory myofibroblastic tumor (IMT) is an uncommon mesenchymal neoplasm composed of myofibroblasts and characterized by an associated inflammatory infiltrate [1]. In general, this neoplasm follows an indolent clinical course; however, in a small percentage of cases, it may exhibit aggressive behavior [2,3]. Common symptoms include abnormal uterine bleeding and pelvic pain, and imaging findings are nonspecific [1,3-6]. The diagnosis is based on histopathologic examination with confirmation by immunohistochemistry and molecular characterization, particularly anaplastic lymphoma kinase (ALK) expression and ALK gene rearrangements [1,6-9]. This report describes a case of uterine IMT occurring in an older woman and discusses the clinical implications and diagnostic criteria associated with this neoplasm.

## Case presentation

A 75-year-old woman presented with heavy abnormal uterine bleeding and hemodynamic instability. Her medical history included hypertension, hypercholesterolemia, atrial fibrillation, vertigo, depression, and Paget’s disease. She had one prior childbirth, with menarche at age 12 and menopause at 51, and no history of hormone replacement therapy.

Transvaginal ultrasound revealed a uterine mass. She subsequently underwent hysteroscopy with biopsy, which showed a small fragment of smooth muscle without atypia. There was no evidence of malignancy.

**Figure 1:**
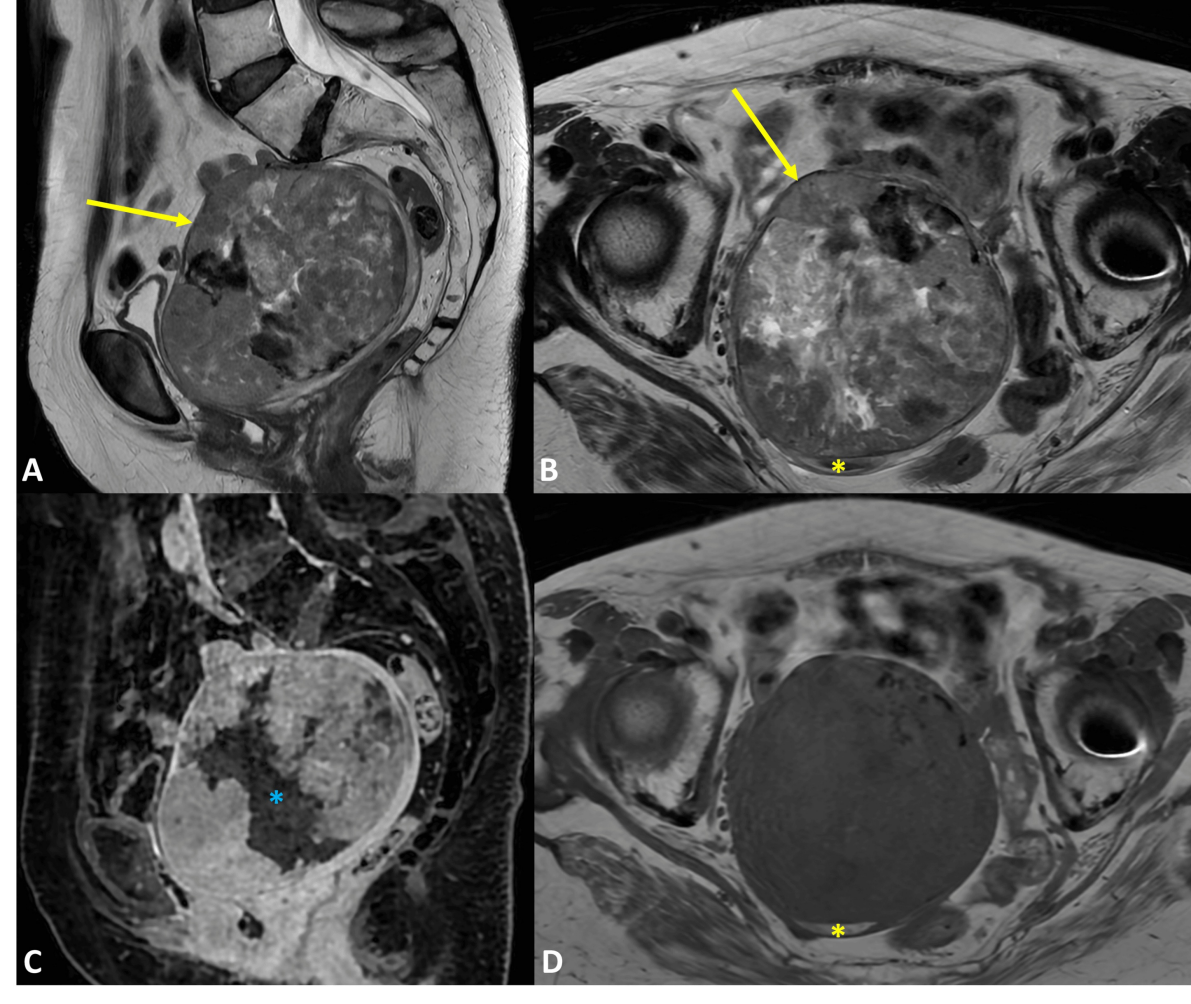
Morphologic MRI sequences of the uterine inflammatory myofibroblastic tumor. Sagittal T2-weighted (A), axial T2-weighted (B), sagittal post-contrast fat-saturated T1-weighted (C), and axial T1-weighted images show a large solid myometrial tumor in the anterior wall of the uterine body (yellow arrow), with intermediate signal and some hypointense areas on T2 images, and regions of chronic hemorrhagic content (yellow asterisk). There is central necrosis (blue asterisk) and no extrauterine extension on the post-contrast fat-saturated T1-weighted image.

**Figure 2:**
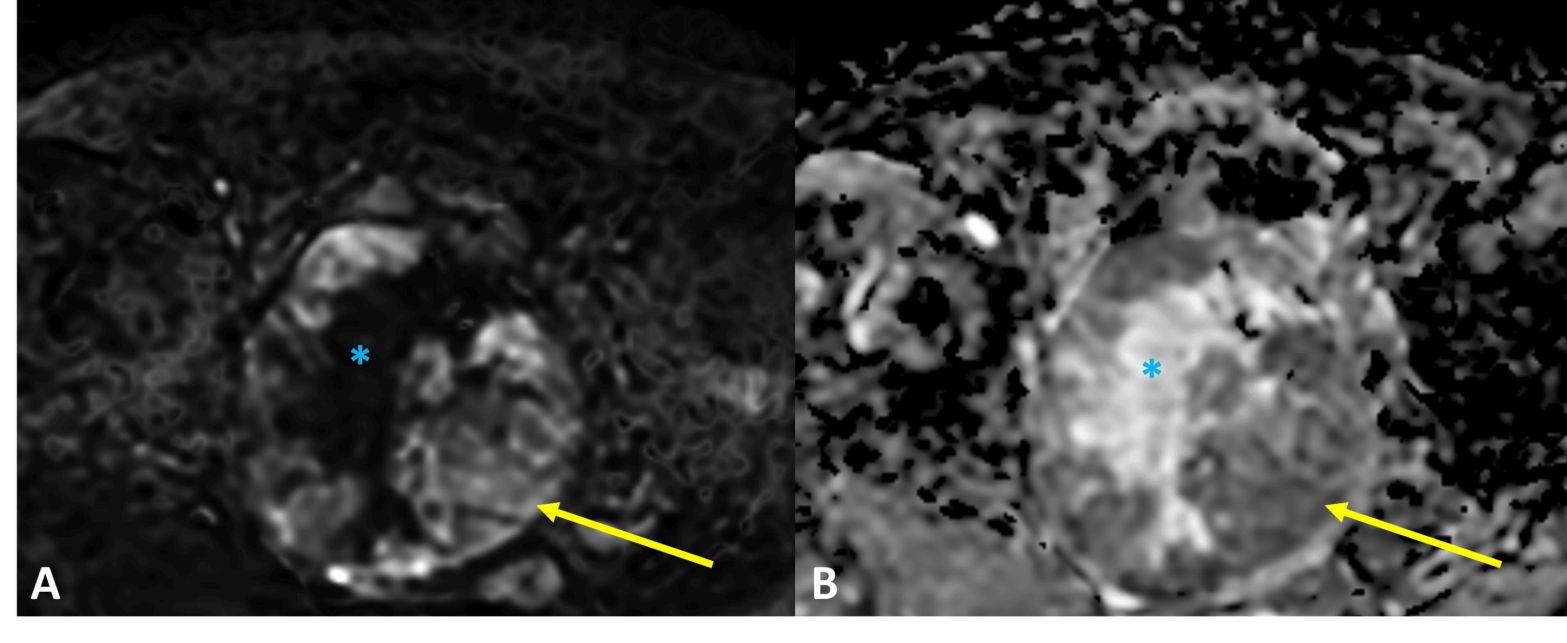
Diffusion-weighted and ADC images of the uterine inflammatory myofibroblastic tumor. Axial diffusion-weighted imaging (DWI) at b = 1000 (A) and the apparent diffusion coefficient (ADC) map (B) show low ADC values in the solid component (yellow arrow), consistent with true restricted diffusion, and higher ADC values centrally (blue asterisk) within the necrotic area shown in Figure 1, consistent with facilitated diffusion.

**Figure 3:**
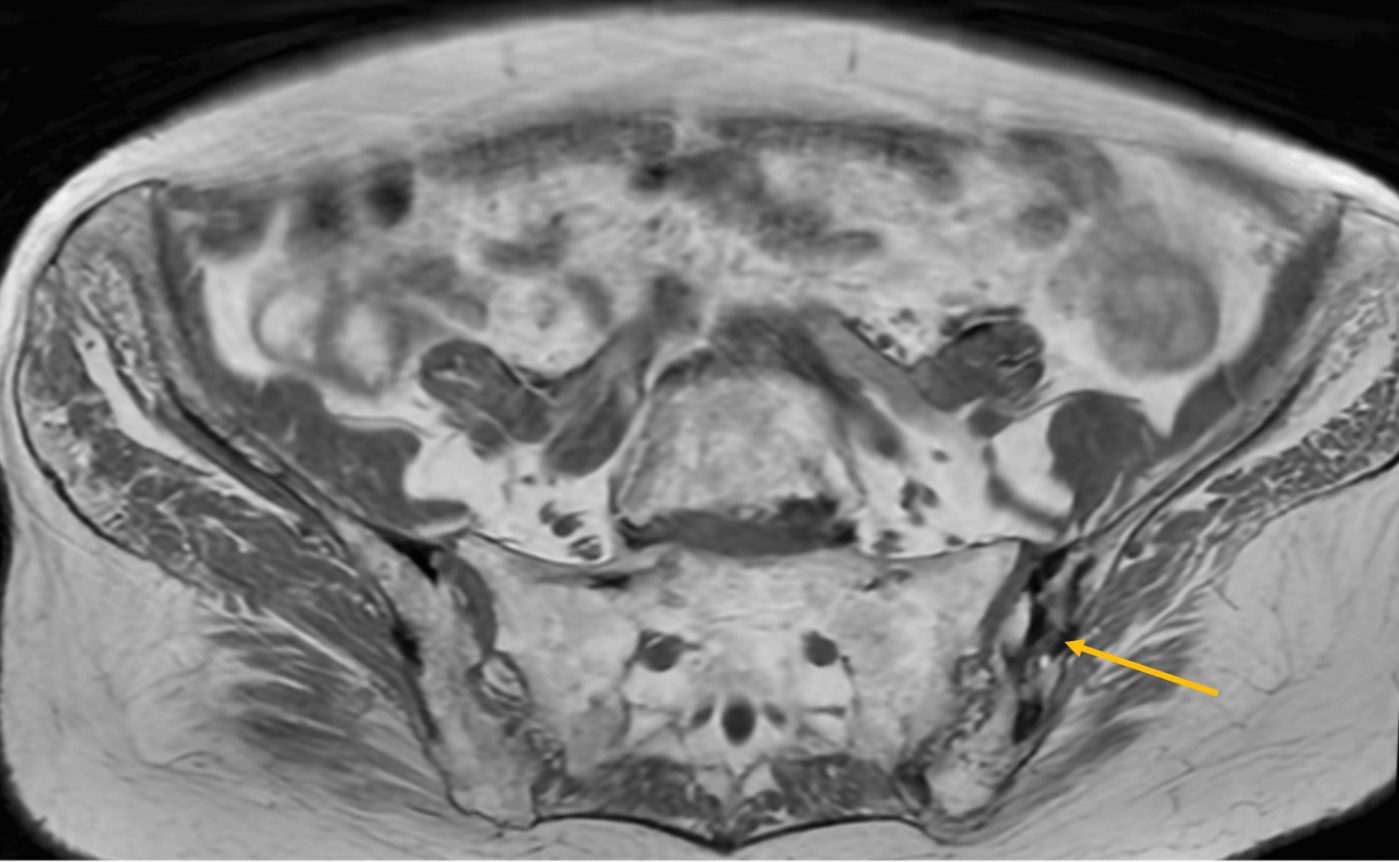
Paget’s disease of the left iliac bone. Axial T1-weighted image shows heterogeneous low signal in the left iliac bone (orange arrow), compatible with the patient’s known Paget’s disease.

Abdominal and pelvic MRI demonstrated a large myometrial tumor in the anterior wall of the uterine body, measuring 11.7 × 9.6 × 9 cm in the longitudinal, anteroposterior, and transverse dimensions, respectively. The tumor showed intermediate signal intensity on T2-weighted imaging, with areas of hypointensity and regions of chronic hemorrhagic content (Figures 1A, 1B, 1D). Post-contrast fat-saturated T1-weighted imaging demonstrated central necrosis without extrauterine extension (Figure 1C). The solid component showed restricted diffusion, suggesting high cellularity (Figure 2). Additionally, bony changes were noted in the left iliac bone, consistent with the patient’s known Paget’s disease (Figure 3).

**Figure 4:**
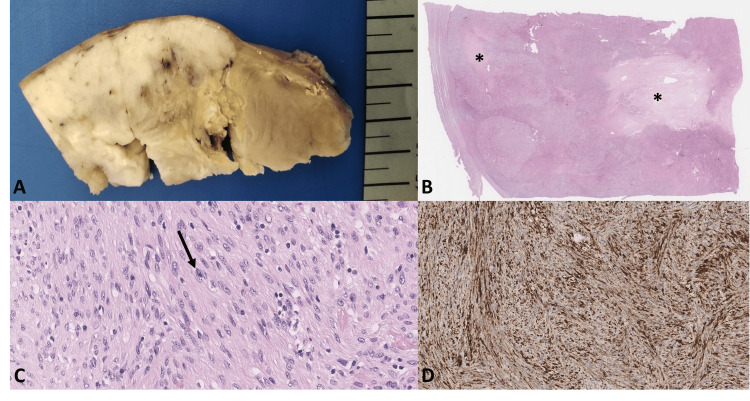
Gross, microscopic, and immunohistochemical findings of the uterine inflammatory myofibroblastic tumor. Gross examination (A) reveals a 10 cm white-yellow mass with approximately 20% necrosis. The tumor infiltrated the myometrium but did not involve the uterine serosa. Microscopically (B), the lesion is a well-circumscribed mesenchymal neoplasm with areas of coagulative necrosis (black asterisk). At high magnification (C), the tumor is composed of spindle-shaped cells exhibiting moderate cytologic atypia. A moderate lymphoplasmacytic inflammatory infiltrate is present, with scattered plasma cells (black arrow). No mitotic figures are identified. Immunohistochemical analysis (D) demonstrates diffuse expression of smooth muscle actin, desmin, and caldesmon. ALK immunostaining is positive. Fluorescence in situ hybridization (FISH) confirms the presence of an anaplastic lymphoma kinase (ALK) gene rearrangement.

Total hysterectomy with bilateral salpingo-oophorectomy was performed. Histologic examination revealed an IMT of the uterus. Areas of necrosis, as well as myxoid and hemorrhagic changes, were present. The neoplasm infiltrated the full thickness of the myometrial wall but did not breach the serosa. No lymphovascular invasion was identified. On immunohistochemistry, the tumor expressed ALK, smooth muscle actin, desmin, and caldesmon. It was negative for CD10, cytokeratins (CAM5.2 and AE1/AE3), estrogen receptor (ER), S100, CD34, cyclin D1, and pan-TRK. ALK gene rearrangement was confirmed by fluorescence in situ hybridization (FISH) (Figure 4).

## Discussion

Uterine IMT is a rare mesenchymal neoplasm of myofibroblasts with an inflammatory infiltrate [1]. It usually has an indolent clinical course, although a minority recur or metastasize [2].

Patients usually present with abnormal uterine bleeding or pelvic pain [1,3]. Occasionally, it can be found incidentally [1].

Imaging findings of uterine IMTs are nonspecific and can mimic leiomyoma variants or leiomyosarcoma [4,5]. Definitive diagnosis typically requires histology with immunohistochemistry and molecular testing [1,4]. Nevertheless, IMTs are usually isointense to slightly hypointense on T1-weighted images and heterogeneously hyperintense on T2-weighted images, depending on their content [3,4,6]. These tumors typically present as a myometrial mass with heterogeneous enhancement, which may be delayed due to the fibrous or myxoid stroma, and may appear either well demarcated or infiltrative [1,3]. Mild to moderate diffusion restriction can be observed, depending on cellularity [3,6]. Necrosis is more frequent in aggressive cases [7]. In our case, the tumor is isointense on T1-weighted images and heterogeneous with intermediate signal and some hypointense areas on T2-weighted images, and demonstrates central necrosis and diffusion restriction. Conversely, leiomyosarcomas more commonly demonstrate irregular or ill-defined margins, regions of necrosis, intratumoral hemorrhage, and marked restricted diffusion [8,9]. Variants of leiomyoma frequently simulate malignancy when they exhibit degeneration or increased cellularity [8].

Histologically, IMTs are characterized by spindle to stellate myofibroblasts in myxoid to compact stroma with lymphoplasmacytic inflammation [1,10]. The cytologic atypia and mitotic activity are variable [2]. Immunohistochemical studies frequently demonstrate ALK positivity and expression of smooth muscle actin, with variable staining for desmin and CD10 [6,10,11]. At the molecular level, ALK gene fusions are common [2,6,10].

A minority of uterine IMTs behave aggressively. Tumors larger than 7 cm, moderate to severe cytological atypia, high mitotic index, necrosis, and lymphovascular infiltration are associated with poorer outcomes [12].

Treatment consists of complete surgical excision. In some cases, these tumors can also be treated with targeted therapy, specifically ALK inhibitors [1,6]. Imaging-based surveillance is warranted given the potential for local recurrence within about two years in aggressive cohorts [7].

## Conclusions

Uterine IMT is an uncommon entity with variable clinical behavior and largely nonspecific imaging features, making preoperative diagnosis challenging. The identification of the histopathological, immunohistochemical, and molecular characteristics of this tumor is valuable in establishing the proper diagnosis and guiding treatment and management. While most uterine IMTs have an indolent clinical course that is effectively managed with complete surgical resection, some require close imaging follow-up. Targeted therapy may be used in selected cases. A uterine IMT should be considered when a “fibroid-like” uterine mass shows myxoid stroma, heterogeneous enhancement, infiltrative margins, or atypical clinical course. Early consideration of this diagnosis may facilitate optimal treatment and surveillance strategies.
